# Structure of the cystathionine γ-synthase MetB from *Mycobacterium ulcerans*

**DOI:** 10.1107/S1744309111029575

**Published:** 2011-08-16

**Authors:** Matthew C. Clifton, Jan Abendroth, Thomas E. Edwards, David J. Leibly, Angela K. Gillespie, Micah Ferrell, Shellie H. Dieterich, Ilyssa Exley, Bart L. Staker, Peter J. Myler, Wesley C. Van Voorhis, Lance J. Stewart

**Affiliations:** a Seattle Structural Genomics Center for Infectious Disease (SSGCID), USA; b Emerald BioStructures, 7869 NE Day Road West, Bainbridge Island, WA 98110, USA; c School of Medicine, University of Washington, Seattle, WA 98195, USA; d Seattle Biomedical Research Institute, 307 Westlake Avenue North, Suite 500, Seattle, WA 98109, USA; e Departments of Global Health and Medical Education and Biomedical Informatics, University of Washington, Seattle, WA 98195, USA

**Keywords:** pyridoxal phosphate, l-methionine, *O*^4^-succinyl-l-homoserine, l-cysteine, l-cystathionine, AAT-I superfamily, *Mycobacteria ulcerans*, cystathionine γ-synthase

## Abstract

Cystathionine γ-synthase (CGS) is a transulfurication enzyme that catalyzes the first specific step in l-methionine biosynthesis by the reaction of *O* ^4^-succinyl-l-­homoserine and l-cysteine to produce l-cystathionine and succinate. Controlling the first step in l-methionine biosythesis, CGS is an excellent potential drug target. *Mycobacterium ulcerans* is a slow-growing mycobacterium that is the third most common form of mycobacterial infection, mainly infecting people in Africa, Australia and Southeast Asia. Infected patients display a variety of skin ailments ranging from indolent non-ulcerated lesions as well as ulcerated lesions. Here, the crystal structure of CGS from *M. ulcerans* covalently linked to the cofactor pyridoxal phosphate (PLP) is reported at 1.9 Å resolution. A second structure contains PLP as well as a highly ordered HEPES molecule in the active site acting as a pseudo-ligand. These results present the first structure of a CGS from a mycobacterium and allow comparison with other CGS enzymes. This is also the first structure reported from the pathogen *M. ulcerans*.

## Abstract 2

Cystathionine γ-synthase (CGS) is a transferase that catalyzes the reaction between *O* ^4^-succinyl-l-homoserine and l-cysteine to produce l-­cystathionine and succinate. The crystal structure of CGS from *M. ulcerans* is presented covalently linked to the cofactor pyridoxal phosphate (PLP). A second structure contains PLP as well as a highly ordered HEPES molecule in the active site acting as a pseudo-ligand. This is the first structure ever reported from the pathogen *M. ulcerans*.

## 1. Introduction

Methionine is an essential amino acid in humans; however, in plants and many microorganisms methionine is synthesized from both aspartic acid and cysteine. As part of this pathway, cystathionine γ-­synthase (CGS; EC 2.5.1.48) catalyzes the reaction between *O* ^4^-succinyl-l-­homoserine and l-cysteine to produce l-cystathionine and succinate. CGS is a transferase and acts in the committed step (the fifth overall) of the biosynthesis of l-methionine. In bacteria this mechanism is performed by the enzyme MetB, which additionally plays roles in both selenoamino-acid metabolism and sulfur metabolism. MetB is covalently linked to the cofactor pyridoxal phosphate (PLP), similar to other members of the aspartate aminotransferase (AAT-I) superfamily of enzymes. Similar to other CGS enzymes, MetB forms a homotetramer, with each individual homodimer creating two active sites (Clausen *et al.*, 2000 ▶).

The first CGS structure was solved for the *Escherichia coli* enzyme covalently linked to the cofactor PLP (Clausen *et al.*, 1998 ▶) and was followed by a structure of CGS from the plant *Nicotiana tabacum* also covalently linked to PLP (Steegborn *et al.*, 1999 ▶). Steegborn and coworkers also solved a series of structures bound to a variety of inhibitors as potential herbicides (Steegborn *et al.*, 2001 ▶). With the exception of the inhibitor complexes, there are no structures of MetB bound to any ligand or ligand-like compound at high resolution.

*Mycobacterium ulcerans* is the third most common form of mycobacterial infection behind *M. tuberculosis* and *M. leprae* and mainly affects people from Africa, Australia and Southeast Asia (Walsh *et al.*, 2010 ▶). Upon infection, this slow-growing mycobacterial infection produces painful ulcerated lesions known as Buruli ulcers. In the early stages of treatment Buruli ulcers can be treated with antibiotics; however, in later stages the ulcers have to be excised and in some cases lead to amputation (Nienhuis *et al.*, 2010 ▶). To prevent or fight this painful disease, it is of interest to obtain structural information about potential drug targets from the organism.

MetB and other PLP-containing enzymes have been identified as prime drug targets for infectious disease organisms such as mycobacteria (Amadasi *et al.*, 2007 ▶). In addition, mycobacteria prefer using methionine as a source of sulfur, adding to the significance of these targets (Wheeler *et al.*, 2005 ▶). Here, we present two structures of the cystathionine γ-synthase MetB from *M. ulcerans* covalently linked to PLP and bound to the buffer molecule HEPES.

## 2. Methods

### 2.1. Protein expression and purification

The full-length *M. ulcerans* cystathionine γ-synthase MetB (CGS) gene encoding 388 amino acids (NCBI YP_904423.1; UniProt A0PKT3; Pfam ID PF01053; EC 2.5.1.48) was amplified from *M. ulcerans* Agy99 genomic DNA using the oligonucleotide primers 5′-GGGTCCTGGTTCGATGAAGGACGATCACAAGGCGC-3′ (forward) and 5′-CTTGTTCGTGCTGTTTATTAGCCCAGCGCC­TGTTTGAGGTC-3′ (reverse) (Integrated DNA Technologies Inc.). MetB was cloned into pAVA0421 vector (Alexandrov *et al.*, 2004 ▶) by ligation-independent cloning (LIC; Aslanidis & de Jong, 1990 ▶) to produce a construct with an N-terminal hexahistidine tag followed by the cleavage sequence for 3C protease (the full expression tag sequence is MAHHHHHHMGTLEAQTQ′GPGS). The protein was expressed in *Escherichia coli* BL21 (DE3) cells in 2 l auto-induction medium (Studier, 2005 ▶) in a LEX bioreactor (Harbinger, Markham, Ontario, Canada) at 293 K for 72 h, after which the harvested cells were flash-frozen in liquid nitrogen. The frozen cell pellet was thawed and resuspended by vortexing in 200 ml lysis buffer [20 m*M* HEPES pH 7.4, 300 m*M* NaCl, 5% glycerol, 30 m*M* imidazole, 0.5% CHAPS, 10 m*M* MgCl_2_, 3 m*M* β-mercaptoethanol, 1.3 mg ml^−1^ protease-inhibitor cocktail (Roche, Basel, Switzerland) and 0.05 mg ml^−1^ lysozyme]. The cell suspension was disrupted on ice by sonication for 15 min with 5 s pulses at 70% amplitude using a Branson 450D Sonifier (Branson Ultrasonics, Danbury, Connecticut, USA). The sonified solution was incubated with 20 µl Benzonase nuclease (EMD Chemicals, Gibbstown, New Jersey, USA) for 40 min at room temperature with gentle agitation. The lysate was clarified by cen­trifugation with a Sorvall RC5 at 10 000 rev min^−1^ for 60 min at 277 K in an F14S Rotor (Thermo Fisher, Waltham, Massachusetts, USA). The clarified solution was syringe-filtered through a 0.45 µm cellulose acetate filter (Corning Life Sciences, Lowell, Massachusetts, USA). The tagged MetB was purified by affinity chromatography using a HisTrap FF 5 ml column (GE Biosciences, Piscataway, New Jersey, USA) equilibrated in binding buffer (25 m*M* HEPES pH 7.0, 300 m*M* NaCl, 5% glycerol, 30 m*M* imidazole, 1 m*M* DTT) and was eluted with 500 m*M* imidazole in the same buffer.

The peak fractions containing MetB were pooled and concentrated. The protein concentration was determined by measuring the absorption at 280 nm. To cleave the N-terminal affinity tag, 3C protease containing an N-terminal His tag followed by maltose-binding protein (Alexandrov *et al.*, 2001 ▶) was mixed with the target in a 1:50 ratio and the mixture was dialyzed overnight at 277 K against cleavage buffer (20 m*M* HEPES pH 7.6, 500 m*M* NaCl, 5% glycerol, 1 m*M* TCEP). Following cleavage, there were four amino acids (GPGS) that remained as cloning artifacts at the N-terminus of the protein. Any uncleaved protein, 3C protease and cleaved tag were removed by subtractive nickel-affinity chromatography.

The dialyzed sample was collected and imidazole was added to achieve a concentration of 50 m*M*. The sample was incubated with 5 ml Ni Sepharose 6 Fast Flow resin (GE Healthcare, Piscataway, New Jersey, USA) for 1 h at 277 K. The sample–resin slurry was loaded into a 20 ml Econo-Pac chromatography column (Bio-Rad, Hercules, California, USA) and subsequently washed with 10 ml wash buffer (20 m*M* HEPES pH 7.0, 300 m*M* NaCl, 40 m*M* imidazole, 1 m*M* TCEP, 5% glycerol); both the flowthrough and the wash were saved. Flowthrough and wash fractions from secondary affinity chromatography were pooled and concentrated using an Amicon Ultra-15 30 kDa molecular-weight cutoff concentrator (Millipore, Billerica, Massachusetts, USA). Size-exclusion chromatography (SEC) was performed using a Superdex 75 HiLoad 26/60 column (GE Healthcare, Piscataway, New Jersey, USA) that also exchanged the protein into crystallization buffer (20 m*M* HEPES pH 7.0, 300 m*M* NaCl, 5% glycerol, 1 m*M* TCEP). Peak fractions were collected and assessed for purity by SDS–PAGE on a 4–20% Pierce Protein Gel (Thermo Fisher) and were visualized by Coomassie staining with InstantBlue colloidal stain (Expedeon, San Diego, California, USA). Pure fractions were pooled, concentrated and flash-frozen in liquid nitrogen. The final concentration was determined by spectrophoto­metry at 280 nm and final purity was assayed by SDS–PAGE. Samples were flash-frozen in liquid nitrogen and stored at 193 K.

### 2.2. Crystallization

Sitting-drop vapor-diffusion crystallization trials were set up at 289 K using the JCSG+ and PACT crystallization screens (Newman *et al.*, 2005 ▶). MetB stock solutions at 37 and 74 mg ml^−1^ (0.4 µl) were mixed with 0.4 µl reservoir solution and equilibrated against 100 µl reservoir solution using 96-well Compact Jr plates from Emerald BioSystems. Crystals grew in several conditions, but those used for X-­ray data collection and structure determination were obtained in JCSG+ condition D9, which consists of 25.5% PEG 4000, 15% glycerol and 170 m*M* ammonium sulfate. The crystals for both structures were typically 0.2–0.3 mm in size and were yellow in color owing to the presence of covalently linked PLP.

### 2.3. Data collection and structure determination

Single crystals of both MetB–PLP and MetB–PLP–HEPES were directly frozen in liquid nitrogen utilizing the 15% glycerol from the crystallization condition as a cryoprotectant. The MetB-PLP data set was collected in-house using a Rigaku SuperBright FR-E+ rotating-anode X-ray generator with Osmic VariMax HF optics and a Saturn 944+ CCD detector (Table 1 ▶). The crystal-to-detector distance was set to 50 cm and data were collected with 0.5° oscillations over 360° with 10 s exposure time. The overall mosaicity was 0.74°, with 118 776 measured intensities and an overall Wilson *B* factor of 16.2 Å^2^. MetB–PLP–HEPES data were collected on Advanced Light Source beamline 5.0.3 using a 3 × 3 CCD array (ADSC Q315R) detector. The crystal-to-detector distance was set to 200 cm and data were collected in 1.0° oscillations over 180° with 5 s exposure time. The overall mosaicity was 0.29°, with 182 652 measured intensities and an overall Wilson *B* factor of 12.4 Å^2^. Both data sets were reduced with *HKL*-2000 (Otwinowski & Minor, 1997 ▶). The structure was solved by molecular replacement with *Phaser* (McCoy *et al.*, 2007 ▶) from the *CCP*4 suite (Winn *et al.*, 2011 ▶) using molecule *B* of XometC from *Xanthomonas oryzae* pv. *oryzae* (54% identity; PDB entry 3nnp; P.-­T. H. Ngo, J.-K. Kim & L.-W. Kang, unpublished work) as the search model. MetB–PLP was initially rebuilt with *ARP*/*wARP* (Langer *et al.*, 2008 ▶), followed by multiple rounds of refinement in *REFMAC*5 (Murshudov *et al.*, 2011 ▶) and manual building in *Coot* (Emsley & Cowtan, 2004 ▶). MetB–PLP–HEPES was isomorphous to the MetB–PLP structure and could be refined directly using the phases from the previously solved MetB–PLP structure in *REFMAC*5 (Murshudov *et al.*, 2011 ▶). The final models contained two homodimers of MetB spanning residues Ala12–Gly388, with each monomer covalently bound to a single PLP moiety. Both structure models showed good geometry and correctness (Table 2 ▶) according to analysis with *MolProbity* (Chen *et al.*, 2010 ▶).

## 3. Results and discussion

### 3.1. Overall structure

**Figure 1:**
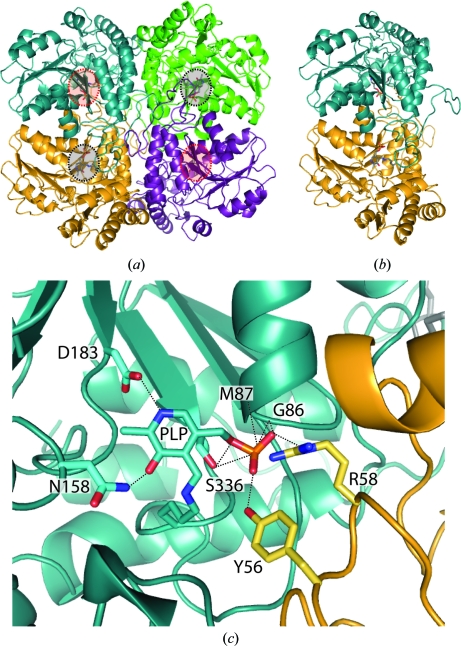
The overall structure of MetB from *M. ulcerans*. (*a*) In both the PLP and the PLP and HEPES complex structures a tetramer was found similar to previous MetB structures. Each tetramer contains two functional dimers (yellow/blue, purple/green). Active sites facing out of the page are indicated in black, while active sites facing into the page are indicated in red. (*b*) An individual dimer of MetB contains two active sites. The main portion of the active site is made up by a pocket within the individual monomer, with a flexible loop from the second monomer closing the pocket. The PLP moiety is shown in gray. (*c*) The active site of MetB covalently linked to PLP. A tight hydrogen-bond network is created around the PLP moiety by residues from both of the individual monomers in the homodimer. Monomer *A* is indicated in blue and monomer *B* in yellow; hydrogen bonds are represented by black dashed lines and range from 2.4 to 3.2 Å in length. The average *B* factor for PLP for the MetB–PLP structure is 14.2 Å^2^.

MetB from *M. ulcerans* has 51% sequence identity to MetB from *X. oryzae* pv. *oryzae* and 40% sequence identity to MetB from *Saccharomyces cerevisiae* after alignment with *ClustalW* (Thompson *et al.*, 1994 ▶). Similar to MetB from *X. oryzae* and *S. cerevisiae*, MetB from *M. ulcerans* is ordered as a homotetramer, with two individual dimers tightly wrapped together to form two active sites per homodimer (Figs. 1 ▶ *a* and 1 ▶ *b*; P.-T. H. Ngo, J.-K. Kim & L.-W. Kang, un­published work; Messerschmidt *et al.*, 2003 ▶). The overall fold of these similar structures is also conserved. Covalently bound at the intersection between the two monomers is the PLP-Lys208 moiety. PLP binds tightly to Lys208 with a covalent-bond length ranging between 1.3 and 1.4 Å. The PLP cofactor is stabilized by a series of hydrogen bonds from Gly86, Met87, Asn158, Asp183 and Ser205 from one monomer and Tyr56 and Arg58 from the second monomer (Fig. 1 ▶ *c*).

### 3.2. Product state

Cocrystallization trials with MetB–PLP and *O* ^4^-succinyl-l-homoserine, l-cysteine, l-cystathionine, succinate and combinations of products and reactants proved unsuccessful, showing only the presence of the PLP covalently bound in the active site. However, the MetB–PLP–HEPES structure suggests that the active site is likely to be blocked to binding of these molecules owing to the presence of a well ordered HEPES molecule. The presence of bound HEPES in the active site of MetB does not change the protein conformation locally or globally (the superposition r.m.s.d.s for MetB–PLP and MetB–PLP–HEPES were 0.134 Å for molecule *A*, 0.136 Å for molecule *B*, 0.151 Å for molecule *C*, 0.141 Å for molecule *D* and 0.15 Å overall calculated on all common C^α^ atoms). The only noted change is seen in a loop consisting of residues 350–362, in which the disordered loop in the MetB–PLP structure becomes partially ordered in the MetB–PLP–HEPES structure.

**Figure 2:**
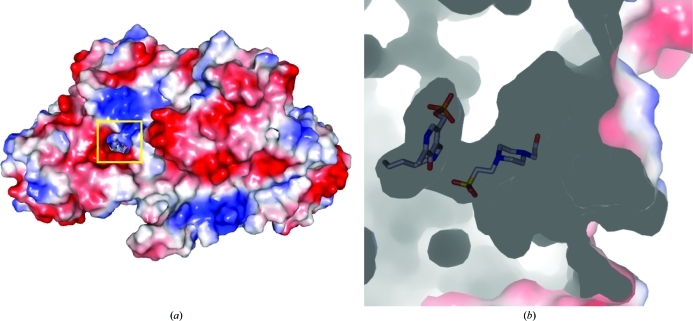
A surface electrostatics representation of MetB–PLP–HEPES. (*a*) The surface shows a small pocket that forms the active site between the individual monomers. (*b*) A side view of the surface representation shows PLP buried deep in the active site and HEPES bound tightly in the position expected for the starting reactant *O* ^4^-succinyl-l-­homoserine.

**Figure 3:**
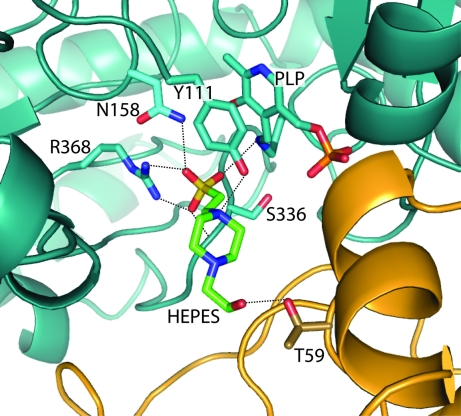
The active site of MetB covalently linked to PLP and bound to HEPES. The tight binding environment for the HEPES molecule is formed by a hydrogen-bonding network created by molecules of both monomers in the homodimer. Monomer *A* is indicated in blue, monomer *B* in yellow and HEPES in green; hydrogen bonds are represented by black dashed lines and range from 2.4 to 3.2 Å in length. The average *B* factors for the PLP and HEPES molecules in the MetB–PLP–HEPES structure are 9.1 and 15.9 Å^2^, respectively.

HEPES is only found ordered in domain *A*; however, all the other monomers contain a well ordered sulfate at this location, suggesting that there may be a poorly ordered HEPES bound in each case (Fig. 2 ▶). There is no apparent conformational change observed for the PLP moiety in the presence or absence of HEPES. A similar series of sulfates are found in the MetB–PLP structure; however, there is no evidence for the presence of HEPES or any other ligand bound in the four active sites. HEPES is bound in the active site with the sulfate head group pointing in towards the pocket and only 3.1 Å from the ∊-­amino group of the PLP–Lys208 moiety (Fig. 3 ▶). The HEPES molecule is bound by a series of hydrogen bonds from the homodimer, including residues Tyr111, Asn158, Ser336, Arg368 and the PLP–Lys208 moiety of one monomer and Thr59 of the second monomer. This tight hydrogen-bond network suggests the approximate position where the starting reactant *O* ^4^-succinyl-homoserine would be bound in the MetB active site to form the initial Michaelis complex (Clausen *et al.*, 1998 ▶).

## 4. Conclusion

**Figure 4:**
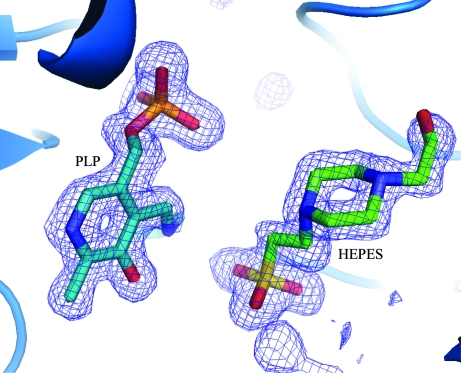
Electron density in the active site of MetB–PLP–HEPES. A 2*F*_o_ − *F*_c_ electron-density map contoured at 3σ (blue) clearly shows the positions of both PLP and HEPES in the binding site of MetB.

We have obtained two high-resolution structures of MetB from *M. ulcerans*, one covalently bound to PLP and one bound to PLP and HEPES, which binds in a similar position to that expected for the starting reactant *O* ^4^-succinyl-homoserine (Fig. 4 ▶). This structure also represents the first reported structure from the organism *M. ulcerans*.

## Supplementary Material

PDB reference: MyulA.00906.a, 3qi6

PDB reference: 3qhx

## Figures and Tables

**Table 1:** Data-collection statistics Values in parentheses are for the highest resolution shell.

Ligands	PLP	PLP and HEPES
Space group	*P*2_1_	*P*2_1_
Unit-cell parameters (Å, °)	*a* = 81.0, *b* = 106.9, *c* = 100.3, β = 113.7	*a* = 80.9, *b* = 106.3, *c* = 100.5, β = 113.7
Wavelength (Å)	1.5418	0.9765
Resolution range (Å)	50–1.91 (1.94–1.91)	30–1.65 (1.71–1.65)
No. of unique reflections	118947 (5238)	182980 (17958)
Multiplicity	6.0 (2.9)	3.8 (3.8)
Completeness (%)	98.1 (86.7)	98.0 (96.5)
*R*_merge_†	0.11 (0.27)	0.09 (0.44)
Mean *I*/σ(*I*)	9.72 (3.70)	8.73 (2.55)

† *R*_merge_ = 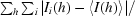 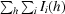.

**Table 2:** Refinement and model statistics Values in parentheses are for the highest resolution shell.

Ligands	PLP	PLP and HEPES
Resolution range (Å)	50–1.91 (1.94–1.91)	30–1.65 (1.71–1.65)
*R*_cryst_†	0.202	0.151
*R*_free_†	0.241	0.182
R.m.s.d. bonds (Å)	0.028	0.027
R.m.s.d. angles (°)	2.09	2.12
Protein atoms	11010	11092
Nonprotein atoms	1625	1542
Mean *B* factor (Å^2^)	16.2	12.4
Residues in favored region (%)	98.0	98.3
Residues in allowed region (%)	1.5	1.1
Residues in disallowed region (%)	0.5	0.6
*MolProbity*‡ score [percentile]	1.56 [93rd]	1.14 [99th]
PDB code	3qi6	3qhx

† *R*_cryst_ = 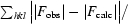 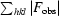. The free *R* factor was calculated using 5% of the reflections omitted from the refinement (Winn *et al.*, 2010 ▶).

‡ Chen *et al.* (2010 ▶).
